# Stimuli‐Induced Subconformation Transformation of the PSI‐LHCI Protein at Single‐Molecule Resolution

**DOI:** 10.1002/advs.202205945

**Published:** 2023-04-28

**Authors:** Zhiheng Yang, Jie Wang, Bing Yin, Wenzhe Liu, Dongbao Yin, Jianren Shen, Wenda Wang, Lidong Li, Xuefeng Guo

**Affiliations:** ^1^ State Key Laboratory for Advanced Metals and Materials School of Materials Science and Engineering University of Science and Technology Beijing Beijing 100083 P. R. China; ^2^ Beijing National Laboratory for Molecular Sciences National Biomedical Imaging Center College of Chemistry and Molecular Engineering Peking University 292 Chengfu Road, Haidian District Beijing 100871 P. R. China; ^3^ Photosynthesis Research Center Key Laboratory of Photobiology Institute of Botany Chinese Academy of Sciences Beijing 100093 P. R. China; ^4^ Center of Single‐Molecule Sciences Institute of Modern Optics Frontiers Science Center for New Organic Matter College of Electronic Information and Optical Engineering Nankai University 38 Tongyan Road, Jinnan District Tianjin 300350 P. R. China

**Keywords:** electric field, photosynthesis, photosystem I‐light harvesting complex I protein, silicon nanowire biosensors, single‐molecule level

## Abstract

Photosynthesis is a very important process for the current biosphere which can maintain such a subtle and stable circulatory ecosystem on earth through the transformation of energy and substance. Even though been widely studied in various aspects, the physiological activities, such as intrinsic structural vibration and self‐regulation process to stress of photosynthetic proteins, are still not in‐depth resolved in real‐time. Herein, utilizing silicon nanowire biosensors with ultrasensitive temporal and spatial resolution, real‐time responses of a single photosystem I‐light harvesting complex I (PSI‐LHCI) supercomplex of *Pisum sativum* to various conditions, including gradient variations in temperature, illumination, and electric field, are recorded. Under different temperatures, there is a bi‐state switch process associated with the intrinsic thermal vibration behavior. When the variations of illumination and the bias voltage are applied, two additional shoulder states, probably derived from the self‐conformational adjustment, are observed. Based on real‐time monitoring of the dynamic processes of the PSI‐LHCI supercomplex under various conditions, it is successively testified to promising nanotechnology for protein profiling and biological functional integration in photosynthesis studies.

## Graphical abstract

A single‐molecule electrical analysis strategy is built to directly decipher the responding reaction dynamics of an individual photosynthetic photosystem I‐light harvesting complex I (PSI‐LHCI) supercomplex for the first time. The responding signals to electric field and illumination show similar patterns, implying that the PSI‐LHCI protein may adopt similar structural adjustment mechanisms.

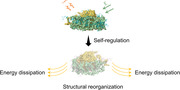

## 1 Introduction

Photosynthesis occupies a decisive position in maintaining the stability of the carbon cycle on earth.^[^ ^1^ ^]^ The robust and astute properties of photosynthetic proteins make an important contribution to the highly efficient photocatalytic capacity. In the photochemical process of photosynthetic organisms, some overexcitation stemming from unpredictable illumination conditions can be subtly coped with the hierarchical self‐regulatory processes, such as the orientation of leaves at the macroscopic level, chloroplast movements at the microscopic level, reconstitution of antenna complexes, and direct energy transfer among chromophores at the nanoscopic level.^[^ ^2^ ^]^ In addition to the detailed analysis of the macroscopic or microscopic aspects of self‐regulation, a thorough understanding of the molecular‐level mechanisms is essential and needs further elucidation.

Nowadays, conformational changes of proteins, rearrangement of pigment molecules, and non‐photochemical quenching of excess energy have already been confirmed to balance light harvesting and photoprotection in photosynthetic proteins.^[^ ^2^ ^]^ Especially, the adjustment of the protein conformations plays a key role in the self‐regulation process under unpredictable conditions.^[^ ^3^ ^]^ However, many details of the mechanism at the nanoscopic or a single molecular level are still controversial or in suspense.^[^ ^2^, ^4^ ^]^ Therefore, directly visualizing the unabridged dynamics of the self‐regulatory process in each protein to elaborate the mechanism at the single‐molecule level is worth exploring.

Photosystem I (PSI) is a well‐known high‐efficiency protein complex for capturing and converting light energy, which contributes to carbon fixation in dark reactions in nature. During photosynthesis, photosystem II utilizes light to split water to generate electrons, which are subsequently transferred by the cytochrome (Cyt) b6/f and PSI to produce nicotinamide adenine dinucleotide phosphate (NADPH), a process known as linear electron transport.^[^ ^5^ ^]^ Furthermore, PSI also uses light to induce photochemical reactions, resulting in a ΔpH gradient that can drive adenosine triphosphate (ATP) synthesis without producing NADPH.^[^ ^6^ ^]^ This process is known as cyclic electron transport which is now thought to be vital for increasing the ATP/NADPH ratio and protecting both photosystems from over‐reduction damage of chloroplast electron carriers.^[^ ^6^, ^7^ ^]^ Whether it is linear or cyclic electron transport, the absorption of light by PSI is an essential step in both processes, enabling the generation and transfer of electrons with an internal quantum efficiency close to unity. In a word, PSI plays a fundamental role in photosynthesis and plant growth.^[^ ^8^ ^]^ As known, this PSI super system is constituted of a reaction center (RC) core and several peripheral light harvesting complex I (LHCI) antennas in alga and higher plants.^[^ ^9^ ^]^ In the photosynthesis process, light energy is captured by pigments in the PSI core and peripheral LHCIs, and then subsequently transferred to the RC to drive electron transfer. Interestingly, the LHCIs and RC have been proven to have good synergy for light adaption. The PSI core with an intact antenna system is more resistant to high light in comparison with the PSI core alone and antenna chlorophyll proteins can function as fuses to protect the photochemical activity of RC.^[^ ^10^, ^11^ ^]^ In addition, the red chlorophylls and *β*‐carotenes in PSI are found sensitive to the applied electric field.^[^ ^12^ ^]^ However, directly recording the response process of the PSI to various illumination intensities and the electric field is still a glamorous challenge to decipher the photoprotection mechanism. Thus, performing a fidelity exploration of the PSI‐LHCI supercomplex under different conditions at the molecular level is also compulsive for an in‐depth understanding of the detailed features of the photoprotection state.^[^ ^11^, ^12^ ^]^

Unlike the results based on the ensemble average, which would conceal characteristics of the individual behavior, some single‐molecule analysis methods, such as fluorescence resonance energy transfer,^[^ ^13^ ^]^ optical or magnetic tweezers,^[^ ^14^ ^]^ surface plasmon resonance,^[^ ^15^ ^]^ atomic force microscopy (AFM),^[^ ^16^ ^]^ scanning transmission electron microscopy (STEM),^[^ ^17^ ^]^ and nanopores,^[^ ^18^ ^]^ have enabled the research of individual dynamics and intermediate structures relying on promoted temporal and spatial resolution. To record the rapid response to light intensity fluctuations occurring in phycobilisomes and light harvesting complex II antennae, single‐molecule spectroscopy has been employed.^[^ ^2^, ^19^ ^]^ The conformational changes, the blink of fluorescence, and fast reversible transitions observed at the single‐molecule level provide great details to describe biological switches between their light‐harvesting and energy‐quenching states. Nevertheless, research on the PSI‐LHCI supercomplex relying on a single molecule method is rarely presented. Apart from high temporal and spatial resolution, the single‐molecule electrical detection techniques represented by single‐molecule junctions (SMJs) and field‐effect transistors (FETs) have stood up with a label‐free advantage that would not obstruct the essential structure information. Furthermore, in situ detections could be performed without interfering with the native function of proteins or DNAs.^[^ ^20^ ^]^

**Figure 1:**
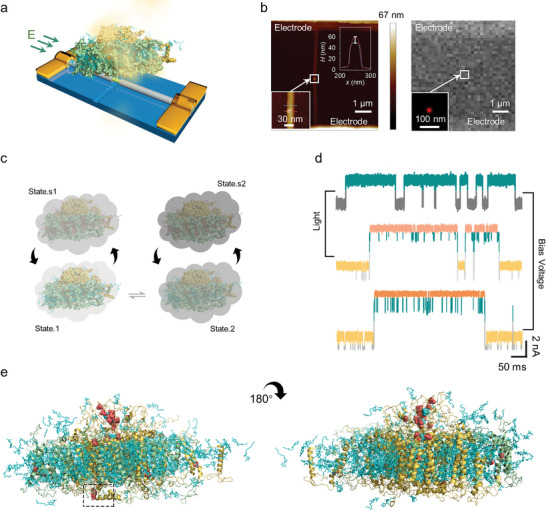
Schematic diagram and characterization of a single‐PSI‐LHCI protein‐modified SiNW FET device. a) Schematic diagram of a SiNW FET device decorated with a single PSI‐LHCI supercomplex (the LHCI antenna domains (green), the pigment and other ligand molecules (cyan), and the PSI domain (yellow) (PBD ID: 4XK8)). b) AFM image of a single PSI‐LHCI supercomplex modified device (left panel). Inset: corresponding magnified part of the marked site, showing a single PSI‐LHCI protein immobilized on the surface of SiNWs. The height of the PSI‐LHCI protein is ≈11.2 nm, consistent with previous reports (≈10.8 nm). Super‐resolution fluorescent image of a single PSI‐LHCI supercomplex modified device (right panel). Inset: a single fluorescent dot image through STORM. c) Schematic diagram of conversion relation among each state of a PSI‐LHCI supercomplex. The grey part represented the surface electron cloud of the PSI‐LHCI supercomplex, and the shade of the color represented electronegativity. d) Real‐time electrical trajectories of a SiNW device regulated by the structural adjustment of the PSI‐LHCI supercomplex responding to different conditions (top: temperature 22 °C; medial: photon flux density 1620 µmol m^−2^ s^−1^; bottom: electric field intensity 0.6 V). e) The potential anchoring site was marked inside the dotted box (natural cysteine sites of the PSI‐LHCI protein) for connection with active bridging molecules in the *cis* and *trans* directions. All Cys residues are indicated in red spheres, among which one site for binding is SiNW labeled by a dashed square.

Recently, 1D silicon nanowire (SiNW) electrical sensors embark on revealing numerous biological mechanisms of enzymatic processes, including protein–protein binding kinetics,^[^ ^21^ ^]^ enzymatic catalysis dynamics,^[^ ^22^ ^]^ and hairpin DNA hybridization kinetics,^[^ ^23^ ^]^ taking advantages of good biocompatibility and high sensitivity. Research on the intrinsic properties of photosynthetic proteins responding to electric fields and illumination has not been reported at a single object level. In this work, to probe the intrinsic properties of photosynthetic proteins, we successfully integrated a single PSI‐LHCI supercomplex, which was separated from higher plant *Pisum sativum*, on the surface of SiNW biosensors, relying on a confined interlinkage. An active maleimide molecule makes a solid bridge with the natural sulfhydryl of cysteine in the PSI‐LHCI supercomplex (**Figure** **1**a,b). Through a series of electrical measurements under various temperatures, illumination, and bias voltage conditions, the vibration behaviors of a single PSI‐LHCI supercomplex under these stress factors, as well as the corresponding structural adjustment in vitro, were unveiled in real time for the first time (Figure 1c,d). As a result of the thermal energy transfer between different parts of the protein molecule, the vibration behaviors are closely dependent on temperature, which can be considered as a thermodynamic model. The extra conformational transformations responding to the change of the photon flux density and the bias voltage follow a similar tendency. Based on the results of control experiments, the structural adjustment of PSI‐Lhca4 related to the Stark effect was proposed. Importantly, this work uncovers the self‐regulation mode of the PSI‐LHCI photosynthetic supercomplex under physiological activity temperature and light conditions at the single‐protein level.

## 2 Results and Discussion

### 2.1 Fabrication of Single PSI‐LHCI Decorated Device

The detailed fabrication process of the modified SiNW device is described in our previous work (Figures S1–S3, Supporting Information).^[^ ^24^ ^]^ In brief, relying on precise high‐resolution electron‐beam lithography and HF etching method, the surface of 1D Si/SiO_2_ core–shell SiNWs was windowed to a ≈10 nm nanogap, which was suitable for a single protein in size and exposed with special Si—H bonds for subsequent reactions. The treated SiNW device was successively embellished with undecynic acid via hydrosilylation, *N*‐hydroxysuccinimide (NHS) via esterification, and maleimide via substitution. The PSI‐LHCI supercomplex was purified relying on the method in the supporting information. Owing to the confinement effect, a single PSI‐LHCI protein was precisely linked to the modified surface of the SiNW device through a Michael addition reaction between maleimide and cysteine of the protein (Figure S4, Supporting Information). To depict a proper linking between the natural cysteine sites of the PSI‐LHCI protein (PDB ID: 7DKZ) and the active bridging molecules without being shielded by the surface amino acids and pigments, a single cysteine on the luminal side is marked with spheres as a potential anchoring site (Figure 1e). The SiNW device was characterized with atomic force microscopy (AFM) and stochastic optical reconstruction microscopy (STORM). A single PSI‐LHCI protein was observed on the surface of SiNWs, which was about 11.2 nm in size and consistent with the reported size of ≈10.8 nm (Figure 1b).^[^ ^25^ ^]^ Through the following measurement of the targeted location above the SiNWs by the STORM instrument, a spot of the phosphorescent signal was captured, which further certified the successful decoration of a single PSI‐LHCI protein again (Figure 1b and Figure S5, Supporting Information).

### 2.2 Investigation of a Single PSI‐LHCI Supercomplex Responding to Temperature Variation

**Figure 2:**
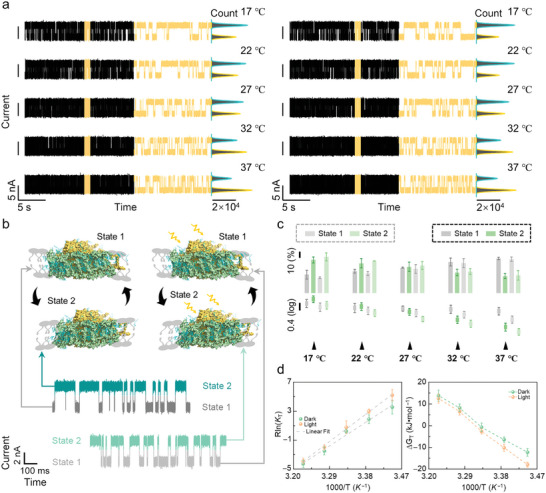
Dynamic analyses of a single PSI‐LHCI protein vibration process in temperature‐dependent experiments. a) Real‐time electrical trajectories of the single PSI‐LHCI protein vibration process under dark (left panel) and light (right panel) conditions (photon flux density 540 µmol m^−2^ s^−1^, bias voltage 0.3 V). Electrical trajectories situated in the middle panel show the 1 s magnified view of each trace and the corresponding current histograms of 20s electrical trajectories are presented on the right. b) Schematic diagram of the vibration process of PSI‐LHCI supercomplex in the bio‐layers under dark and light conditions at 22 °C, photon flux density 540 µmol m^−2^ s^−1^ and bias voltage 0.3 V. c) The distributions of occurrence (top) and dwell time (bottom) responded to each state in temperature‐dependent experiments under dark (dark dashed box) and light (grey dashed box) conditions. d) Thermodynamic statistical plots of the vibration process from the low energy state to the high energy state (Left panel: linear fitting of the value of enthalpy and entropy, Right panel: changes of Gibbs free energy (Δ*G*). mean of *n* = 3 technical replicates from three different single‐PSI‐LHCI‐modified SiNW devices, error bars indicate s.d.

The current signal of a single PSI‐LHCI protein decorated SiNW device was traced under a high‐speed sampling rate (57 600 Sa s^−1^) and a constant source‐drain bias voltage (0.3 V) in a buffer solution (20 mm Tricine‐Tris pH = 7.8). We first conducted temperature‐dependent experiments under both dark and illuminated conditions, with 5 °C intervals ranging from 17 to 37 °C. In the meantime, the preliminary control experiment of the SiNW device without the decoration of the PSI‐LHCI protein was monitored under the same conditions (Figure S6, Supporting Information). In contrast to the mono state observed in the control group with the bare device, a bi‐state transformation emerged in the experimental group (**Figure** **2**a,b). With the increment of temperature, the transformation frequency became faster. The increase in current value of the modified SiNWs compared to the bare SiNWs certified the successful binding of the negatively charged PSI‐LHCI protein with SiNWs (the theoretical isoelectric point of the PSI‐LHCI protein is about 6.81) in the buffer (pH = 7.8). This is due to the charge transport mechanism of a p‐type SiNW,^[^ ^23^ ^]^ where the binding of the negative PSI‐LHCI protein led to an increase in the cave density inside the SiNW. The conductance conversion caused by the structural changes of a single PSI‐LHCI protein was also reproduced on another device (Figure S7, Supporting Information), demonstrating the reproducibility. In addition, the discrepancy in conductance between the two states indicated that State 2 (high conductance state) had a more negative electric property than State 1 (low conductance state) shown in Figure 1c. According to the statistical analysis of the dwell time, the fitting functions of the dwell time of these two states both showed a single exponential decay, indicating that i) there were independent stochastic processes with a constant average rate (Figures S8 and S9, Supporting Information); ii) The values of the dwell time showed a gradual descent with the temperature increment; iii) The occurrence of State 1 increased with the temperature rising at the expense of the occurrence of State 2 (Table S1, Supporting Information, and Figure 2c). These results indicated that the transformation between the two states presented a thermodynamics‐dependent relation, and the conformation of State 1 exhibited a preference for lower temperatures, which is different from State 2.

In a different environment, protein molecules undergo thermal vibrations, which are the result of the transfer of thermal energy between different parts of a protein molecule. At higher temperatures, the increase of the thermal energy of the protein leads to the rapid movement of the constituent atoms and fluctuations in bond length and angle, resulting in protein conformational changes.^[^ ^26^ ^]^ Therefore, the protein molecular flexibility is vital to adapt to a range of temperatures, which influences the mobility of secondary structural components of protein and operates on µs–ms scales instead of the ps‐scale.^[^ ^27^ ^]^ According to our results, there were two states of a single PSI‐LHCI within the range of the physiological activity temperature on µs–ms scales. So, the thermodynamics‐dependent conversion between two states should originate from the intrinsic flexible vibration action of a PSI‐LHCI protein.

Relying on the thermodynamic relationship, −Δ*G*_T_/*T =* Rln*K*_T_ = −Δ*H*_T_/*T*+Δ*S*_T_, where Δ*H*_T_ is the change of enthalpy, which is equivalent to the difference of the total bond energy; Δ*S*_T_ is the change of entropy, which represents the order degree index of the system; *K*_T_ = *P*_2_/*P*_1_, *P* is the value of occurrence corresponding to each state. The entropy change (Dark: −138.27 ± 4.21 J K^−1^ mol^−1^; Light: −120.60 ± 7.29 J K^−1^ mol^−1^) and the enthalpy change (Dark: −41.66 ± 1.26 kJ mol^−1^; Light: −36.11 ± 2.19 kJ mol^−1^) from State 1 to State 2 were obtained through linear fitting between Rln(*K*_T_) and 1000/*T*. These results imply that the transformation from State 1 to State 2 goes along with the increment of the system disorder and bond energy (Figure 2d).^[^ ^28^ ^]^ The change tendency of Gibbs free energy with increasing temperature suggests that the transformation from State 1 to State 2 is initially driven by the internal energy of the PSI‐LHCI protein system and then the external energy (Figure 2d).^[^ ^29^ ^]^

### 2.3 Investigation of a Single PSI‐LHCI Supercomplex Responding to Illumination Variation

**Figure 3:**
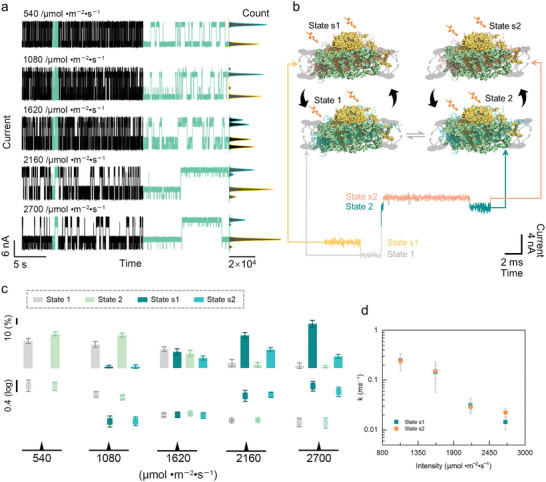
Dynamic analyses of a single PSI‐LHCI protein structural adjustment process in photon flux density‐dependent experiments. a) Real‐time electrical trajectories of the single PSI‐LHCI protein structural adjustment process in photon flux density‐dependent experiments (bias voltage 0.3 V, temperature 22 °C). Electrical trajectories situated in the middle panel show the 1 s magnified view of each trace and the corresponding current histograms of 20 s electrical trajectories are presented on the right. b) Schematic diagram of the structural adjustment process of the PSI‐LHCI supercomplex in the bio‐layers at 22 °C, photon flux density 2700 µmol m^−2^ s^−1^ and bias voltage 0.3 V. c) The distributions of occurrence (top) and dwell time (bottom) responding to each state in photon flux density‐dependent experiments. d) The tendencies of *k* responding to the change of the photon flux density. mean of *n* = 3 technical replicates from three different single‐PSI‐LHCI‐modified SiNW devices, error bars indicate s.d.

To investigate the natural physiological process and the underlying photoprotection mechanistic of PSI‐LHCI in response to illumination, we examined the structural change under gradient illumination conditions from 540 to 2700 µmol m^−2^ s^−1^ with 540 intervals. The current trajectories are exhibited in **Figure** **3**a. Intriguingly, when the photon flux density increased to 1080 µmol m^−2^ s^−1^, two additional shoulder states (State s1 and State s2), whose conductance was higher than those of the corresponding base states (State 1 and State 2), were generated with conversion only occurred between two base states. The current value of base states remained unchanged in comparison with the temperature gradient experiments, which illustrates that the base states should be still ascribed to the thermal vibration structure, while the shoulder states are the associative architecture of the base structures (Figure 3b). Furthermore, the emerging conductance conversion between the base state and shoulder state certifies that some configuration adjustment occurs in the PSI‐LHCI supercomplex.^[^ ^30^ ^]^ The single exponential fitting of the dwell time of each state is shown in Figure S10, Supporting Information, and the distributions of the dwell time and occurrence corresponding to each state are depicted in Figure 3c. Notably, the occurrence and dwell time of shoulder states increased gradually and almost dominated the main structure of the PSI‐LHCI at the photon flux density of 2700 µmol m^−2^ s^−1^ (Table S1, Supporting Information). The dominance of shoulder states over the base states (occurrence: State s1–73.84%, State s2–19.74%, State 1–4.19%, State 2–2.23%) indicated that at this light intensity, a structural adjustment saturation status has almost been reached with respect to a single PSI‐LHCI protein. The velocity constants of each structural adjustment conformation were calculated based on the formula *k* = 1/*τ*, where *τ* is equivalent to the value of the dwell time of each state. The distributions of *k* belonging to two shoulder states at illumination gradient experiments are shown in Figure 3d. The tendency of *k* manifests that the structural adjustment action by structural reconstitution could be remarkably affected by illumination.

**Figure 4:**
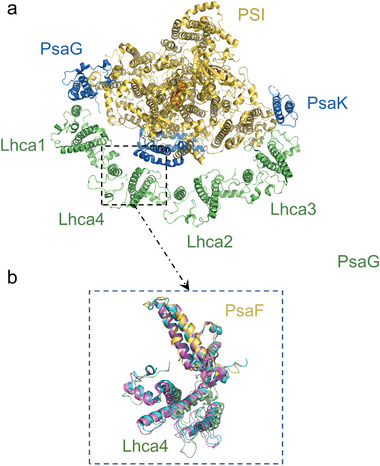
Alignment model of lhca4 and PsaF domains. a) The structural cartoon model of the PSI‐LHCI protein (7DKZ) and b) the alignment model of lhca4 and PsaF domains, which reflected a certain displacement emergence between lhca4 and Psaf domains (Magenta: 5L8R, Sky blue: 7DKZ, Yellow: 4XK8).

Taking the biological function of the PSI‐LHCI supercomplex into consideration, the light can be absorbed by pigments in the peripheral LHCIs and converted into energy, which is subsequently transferred to the RC for electron transfer. However, the efficiency of energy transfer from Lhca2 and Lhca3 to the RC might not be as high as that from Lhca1 and Lhca4, as suggested by the difference in distance between these antennas and the core chlorophylls.^[^ ^31^ ^]^ Some studies show that the energy absorbed by Lhca1 might be transferred completely to the red chlorophylls of Lhca4.^[^ ^32^ ^]^ All of these make Lcha4 an important subunit for PSI‐LHCI to adjust to the light, consistent with the structural properties. The way of combination between LHCI and the PSI core is asymmetrical. The PsaG and PsaK in the core are bound more tightly with Lhca1 and Lhca3 respectively than any other antennas with the core. Lhca4 binds to PsaF only via ionic interactions with a large gap between them.^[^ ^33^ ^]^ Meanwhile, it is worth noting that Lhca4 is more flexible as it can move further from the PSI core when comparing our PSI‐LHCI structure with the formerly published structures (**Figure** **4**).^[^ ^34^ ^]^ Also, some study shows that LHCI would undergo conformation switches to realize its specific functions.^[^ ^35^ ^]^

So, the two new states that emerged under the light conditions in our results are possibly generated by the structural adjustment between Lhca4 and PSI‐core. In addition, in terms of the p‐type SiNW, the increment of the conductance of shoulder states in comparison to the base states also certifies that the enhancement of the electronegativity might originate from the increment of the charge density generated from the activated pigments after absorbing light. In this process, we propose that the pigment molecules in LHCI are excited to high energy states by illumination, and in order to deliver the energy to the core more efficiently, Lhca4 can moderate the distance between it and the core.^[^ ^36^ ^]^ In summary, the illumination gradient experiments have demonstrated the existence of self‐structural adjustment within the PSI‐LHCI supercomplex. This structural adjustment allows the complex to respond to changes in illumination and regulate the distribution of electrical energy levels within LHCI, as well as ensure the healthy operation of the PSI core.

### 2.4 Investigation of a Single PSI‐LHCI Supercomplex Responding to Bias Voltage Variation

**Figure 5:**
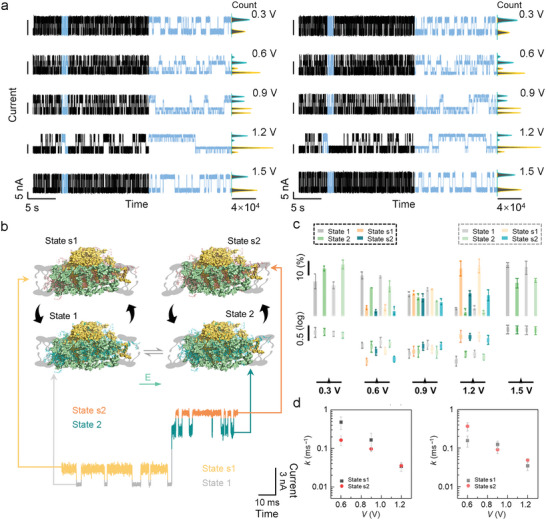
Dynamic analyses of a single PSI‐LHCI protein structural adjustment process in bias voltage‐dependent experiments. a) Real‐time electrical trajectories of the single PSI‐LHCI protein structural adjustment process under dark (left panel) and light (right panel) conditions (photon flux density 540 µmol m^−2^ s^−1^, temperature 22 °C). Electrical trajectories situated in the middle panel show the 1 s magnified view of each trace and the corresponding current histograms of 20 s electrical trajectories are presented on the right. b) Schematic diagram of the structural adjustment process of the PSI‐LHCI supercomplex in the bio‐layers under dark conditions at 22 °C, photon flux density 540 µmol m^−2^ s^−1^, and bias voltage 1.2 V. The green arrow represents the direction of the electric field. c) The distributions of occurrence (top) and dwell time (bottom) responded to each state in bias voltage‐dependent experiments under dark (dark dashed box) and light (grey dashed box) conditions. d) The tendencies of *k* under dark (left panel) or illumination (right panel) conditions respond to the change of bias voltage. mean of *n* = 3 technical replicates from three different single‐PSI‐LHCI‐modified SiNW devices, error bars indicate s.d.

Based on the Stark effect that the pigment molecular energy levels can be perturbed by an external electric field,^[^ ^37^ ^]^ we also measured the response of the PSI‐LHCI supercomplex to an electric field (**Figure** **5**a). The changes in bias voltage also induced another two similar shoulder states (State s1 and State s2) after the bias voltage reached 0.6 V (Figure 5b), and these shoulder states progressively emerged and faded away along with the augment of bias voltages. In addition, control experiments certified that the response trend under electrical field gradients was consistent with that of the response under photon flux density gradients, suggesting that the shoulder states induced by the illumination and electric field have uniform structures and a consistent response mechanism of PSI‐LHCI to both illumination and electric field (Figure S11, Supporting Information). The distributions of the dwell time and occurrence responding to each bias voltage condition are shown in Figure 5c and the single exponential fitting functions of the dwell time belonging to each state are listed in Figures S12 and S13, Supporting Information. The occurrence and dwell time of the base states decreased gradually but were restored at a 1.5 V bias voltage. Inversely, the occurrence and dwell time of shoulder states aggrandized gradually but disappeared at a 1.5 V bias voltage (Table S1, Supporting Information). The conversion between two base states still existed in bias voltage gradient experiments, suggesting that a high bias voltage does not affect the thermal vibration mode of the PSI‐LHCI supercomplex.

Under the bias voltage at 0.9 V, the proportions of occurrence and the dwell time belonging to shoulder states and base states switched. The shoulder state occupied the dominant structure of the PSI, indicating that the structural adjustment behavior performs an increasing function. Until 1.2 V, the occurrence (dark: State 1–9.07%, State s1–58.01%, State 2–5.22%, State s2–27.70%; light: State 1–9.94%, State s1–60.68%, State 2–4.34%, State s2–25.04%) and dwell time (dark: State 1–2.17 ± 0.25 ms, State s1–29.37 ± 4.70 ms, State 2–4.48 ± 0.21 ms, State s2–28.03 ± 4.14 ms; light: State 1–4.68 ± 1.82 ms, State s1–28.81 ± 3.01 ms, State 2–3.28 ± 0.56 ms, State s2–20.48 ± 3.89 ms) of shoulder states arrived the maximum values, suggesting that the structural adjustment mechanism has reached an almost saturated extent. The special features, such as the discrepancy of thermodynamics motif parameter, that is, occurrence, corresponding to two base states and the disappearance of shoulder states at 1.5 V bias voltage, are considered as a result of a permanent structural injury caused by extreme stress conditions. This highlights the limitation of structural adjustment under electric field conditions. Similar electro‐coupled structural damage by the electric field on the structure of membrane protein has been founded in other works.^[^ ^30^, ^38^ ^]^ In this case, because of the degradation of antenna chlorophyll‐proteins, the structural injury blocks the structural adjustment in the PSI‐LHCI supercomplex and the thermal vibration becomes the remaining conformation conversion mode. In addition, two branches of the electron transfer path in the PSI core could also be broken down under strong electric field conditions.^[^ ^36^, ^39^ ^]^

In general, the transition between State 1 and State 2 of PSI‐LHCI under various conditions is attributed to the thermal vibration behavior, as the dynamics of the transition process show a strong dependence on temperature changes. State s1 and State s2 can be induced by both electric field and illumination. The occurrence proportions shift gradually from base states to shoulder states with the increase of the electric field strength and the illumination. Additionally, these two conditions have similar effects on the kinetic parameter (*k*) of the self‐adjustment behavior, indicating the existence of a consistent response mechanism. This consistency was also confirmed by the control experiments. It is reasonable that the PSI‐LHCI supercomplex is sensitive to both electric and light as a highly efficient photoelectric conversion apparatus. But there is still a saturated situation where self/external regulation is required. It is hypothesized that when the PSI‐LHCI supercomplex operates in a proper condition, the absorbed energy could be converted to photochemical energy, transforming PSI‐LHCI into different sub‐conformations for specific functions.^[^ ^3a^ ^]^ No matter the influence of the photon flux density or the electric field strength, both results demonstrate that there is a structural adjustment in the PSI‐LHCI supercomplex. This behavior may be associated with the principal mechanism of the self‐regulation process, which is worth studying in the future to elucidate more details of pigments and residues in the switching process of adjustment of LHCI antennas. It should be mentioned that the applied electric field in our work was not in the physiological direction between internal and external membranes, which will be optimized by regulating the orientation of the protein or the electric field for a more similar electric field environment in the physiological state.

## 3 Conclusion

The visualization of the structural adjustment process in a photosynthetic supercomplex at the single‐molecule level is realized utilizing ultrasensitive SiNW FET biosensors. Remarkably, the thermodynamic vibration behavior and the structural changes during self‐adjustment of the photosynthetic PSI‐LHCI supercomplex responding to an electric field and light were recorded simultaneously in real‐time. The thermodynamic vibrations under various temperatures, electric fields, and light conditions presented regular conversions between low energy levels and high energy levels of PSI‐LHCI, which are consistent with the structural changes of the PSI‐LHCI protein under different conditions. Furthermore, the capacity of the structural adjustment of the PSI‐LHCI supercomplex shows a close relation to the changes in the electric field strength and the photon flux density. Therefore, this work demonstrates flexible and tunable characteristics of PSI‐LHCI contributing to the regulation of pigment molecular energy levels at the single‐molecule level for the first time and also proves that PSI‐LHCI is a high‐performance photoelectric apparatus that is able to adapt to the changes of light and electric conditions. On one hand, along with the improvement of the temporal resolution in the future, that is, femtosecond and picosecond scales, more insights into the photosynthesis mechanism would be revealed by single‐protein devices. On the other hand, new crucial techniques are expected to couple with SiNW FET biosensors to study or develop artificial photosynthetic devices for utilizing solar energy.

## 4 Experimental Section

### The Device Fabrication Process

The detailed growth process of SiNWs is according to the method, which is described in the previous works.^[^ ^20^, ^23^, ^40^ ^]^ The silicon wafers with a 1000 nm‐thick thermally‐grown oxide layer were clipped to a certain size, which was suitable for the function as growth substrates. Then, the gold nanoparticles with an average diameter of 20 nm (Ted Pella) were selected as catalysts dispersing on the surface of substrates. Boron‐doped p‐type SiNWs were synthesized at 470 °C, relying on 2.5 sccm disilane as a reactant source (Matheson Gas Products, 99.998% Purity) and 0.11 sccm diborane as the p‐type dopant (100 ppm, diluted in H_2_) for about 20 min under the condition of a B/Si ratio of 1/100 000 and 7.5 sccm H_2_ as the carrier gas. The synthesized SiNWs were characterized by a scan electron microscope and optical microscope (Figure S1, Supporting Information). SiNWs were transferred to the surface of the 1.4 cm × 1.8 cm designed region of silicon wafers through the mechano‐sliding method.^[^ ^24^ ^]^ Utilizing the accurate photolithographic technique, the SiNWs between the electrodes were protected by a positive resist (ARP 5350) stripe, and the redundant SiNWs were subsequently removed by a feat of sonication. Then, the protective resist was washed off with acetone, and electrode patterns were defined by a standard UV lithography (BG‐401A, China electronics technology Group Corporation). Afterward, the oxide shell of the nanowires was removed through immersion of the predefined silicon wafer into the buffered HF solution (40% NH_4_F:40% HF, 7:1) to generate better Ohmic electrical contacts with metal electrodes. The silicon wafers were immediately attached to the stage of a vacuum coating equipment and 8 nm Cr and 80 nm Au were successively deposited on the silicon wafer to form metal electrodes by thermal evaporation (ZHD–300, Beijing Technol Science). Subsequently, a 40 nm thick SiO_2_ protective layer was deposited on the surface of the electrodes utilizing electron beam thermal evaporation (TEMD–600, Beijing Technol Science) to passivate the contact interface. After the lift‐off of the spare photoresist with copious acetone, the SiNW FET arrays were obtained. Because the testing condition for drain current was constructed inside the buffer solution, a location window between electrodes was generated using the negative resist (SU–8, 2002) by photolithography to protect the major surface of the device.

### Electrical Characterization of SiNW FETs

Utilizing an integrated apparatus consisting of an Agilent 4155C semiconductor analyzer and a Karl Süss (PM5) manual probe station, the electrical characterization of the SiNW FET device was carried out using the heavily doped Si substrate as the global back gate. The good Ohmic contacts of typical p‐type behaviors are represented in Figure S3, Supporting Information.^[^ ^24^, ^41^ ^]^

### Purification of PSI‐LHCI Supercomplex

The purification of peas (*P. sativum* var. Alaska) thylakoid samples was the same as described previously.^[^ ^25^ ^]^ Thylakoids were solubilized in a storage buffer (0.3 m sucrose, 20 mm Tricine pH 8.0) at a concentration of 2.6 Chl mL^−1^, together with 0.55% (w/v) *β*‐DDM for 12 min in ice. Then, added cold water to dilute the solution, centrifuged at 20 000 × *g*, for 10 min, collected the supernatant, and centrifuged at 150 000 × *g* for 30 min. The pellet was suspended in the storage buffer at a concentration of 3 mg Chl mL^−1^ and re‐solubilized with 1.8% (w/v) *β*‐DDM for 15 min in ice. After centrifugation at 150 000 × *g* for 15 min, the supernatant was applied to a DEAE‐650 M column, which was eluted by a 50–250 mm NaCl linear gradient. The PSI‐LHCI was eluted at 180–230 mm NaCl and collected by centrifugation at 15000 × *g* for 5 min after the addition of 10% PEG 6000 (Sigma‐Aldrich). The re‐suspended PSI‐LHCI was solubilized with 0.05% dodecyl‐*β*‐D‐thiomaltopyranoside (*β*‐DDTM) at 1 mg Chl mL^−1^ and applied onto a 0.3–0.9 m sucrose density gradient, followed by centrifugation at 230 000 × *g* for 18 h. Finally, the darkest green band was collected and centrifuged at 15 000 × *g* for 15 min after the addition of 50 mm ammonium acetate and 10% PEG6000. The final pellet suspended in 20 mm Tricine‐Tris (pH 7.5) was the final sample.

### The Immobilization Process of a Single PSI‐LHCI Protein

A polymethyl methacrylate (PMMA 950, A4) layer was covered on the surface of the SiNW FET device by spin coating and then baked at 180 °C for about 2 min. The precise high‐resolution electron beam lithography with a designed CAD file including a ≈10 nm‐wide solid line was employed to open a window precursor at the potential position. After the development for about 1 min at 4 °C in a mixture of water/isopropanol (V: V = 1:3) with the help of sonication, a certain nanogap that was suitable for the accommodation of a single protein in size was formed. Subsequently, the SiNW FET device underwent wet etching in a buffered HF solution (HF (40%): NH_4_F (40%) = 1:7) for about 7 s to corrode the amorphous SiO_2_ shell and show the silicon surface terminated with Si—H bonds inside the certain nanogap. After that, the outdated PMMA layer was cleaned up with acetone. The surface of the SiNW FET device was successively modified to realize the special immobilization of the PSI‐LHCI supercomplex at the single‐molecule level. In order to improve the single‐molecule modification yield, reactive reagents at each step were chosen with high purity. First, the freshly‐etched device was placed inside the bottom of the Schlenk bottle and then covered with 3 mg powders of undecynic acid. After the heating reaction at 90 °C for about 10 h under the regulation of the argon atmosphere, the device was washed with copious dichloromethane to remove reductant unreacted residues above the device and dried with a stream of N_2_. Second, the dried device was immersed into a mixture solution including 1‐ethyl‐3‐(3‐dimethylaminopropyl) carbodiimide (EDC) (10 mm) and NHS (20 mm) for 4 h at ambient temperature to achieve the efficient esterification of the carboxyl group with NHS (pH = 6.5). Then, the modified device was thoroughly washed with deionized water and immediately dried with a stream of N_2_ gas. Third, the device was soaked into an *N*,*N*‐dimethylformamide (DMF) solution which involved a certain concentration of N‐(2‐aminoethyl) maleimide hydrochloride (18 mm) to produce a substituted reaction between the activated carboxyl group and maleimide. The device was subsequently washed with DMF thoroughly and dried with a stream of N_2_ gas. Finally, the 4.5 µm PSI‐LHCI supercomplex was completely dissolved into 10 mm PBS, which contained 20 mm Tricine, 5 mm MgCl_2_, 5 mm KCl, and 0.03% *n*‐dodecyl‐*β*‐D‐maltopyranoside (DDM) at pH = 7.8. A 50 µL PSI‐LHCI supercomplex solution was extracted to overlay the surface of the device for a sufficient reaction for about 24 h at 4 °C and then the redundant PSI‐LHCI supercomplexes were removed by thoroughly washing with buffer solution (Figure S4, Supporting Information). Detailed characterization analyses of each reaction step are executed in the previous work.^[^ ^23^ ^]^

### Real‐Time Electrical Measurements

A PDMS cube opened with a ≈2mm (diameter) hole as a reaction chamber was covered on the surface of a single PSI‐LHCI decorated device. The microchamber was fulfilled with 50 µL buffer solution. The testing temperature was controlled by the INSTEC hot/cold chuck which integrated with a proportion‐integration‐differentiation control system (± 0.001 °C) and a liquid nitrogen cooling system. When the device was set into the platform and the electrode couples were caught with probes, the source‐drain and gate biases were applied at DC 300 and 0 mV, respectively. Relying on an HF2LI Lock‐in Amplifier (Zurich Instruments), the electrical measurements were produced in all the real‐time. Then, a DL1211 preamplifier was utilized to amplify the source‐drain current through a selected SiNW device operating at 10^7^ V A^−1^ gain. The electrical testing data were collected through the HF2LI Lock‐in Amplifier with a bandwidth of 5 kHz low‐pass filter at sampling rates of 57.6 or 7.2 kHz.

### Photon Flux Density

During the electrical detection process in the photon flux density gradient experiments, the photon flux density of the testing environment was detected by an LI‐250A Light Meter, whose wavelength limit ranged from 400 to 700 nm. The button “ON” of the LI‐250A Light Meter was pressed for 2 s, and then the prober was placed on the surface of the device. After the reading was displayed, the button “AVG” was pressed to accumulate a 15‐s average.

### Statistical Analysis

Relying on a low‐pass Butterworth filter with a frequency of 2 kHz, the raw data were used to reduce the signal noise of the circuit. Through the hidden Markov model, the filtered data were then idealized to obtain the dwell time of each signal event and the number of total events.^[^ ^42^ ^]^ The idealized data were extracted as text and imported into Origin 9.0. Through the single‐exponential fitting of the counts and dwell time corresponding to each state, the average dwell time was obtained. Data are presented as mean ± SD. Mean of *n* = 3 technical replicates from three different single‐PSI‐LHCI‐modified SiNW devices, error bars indicate s.d. Statistical analysis was carried out using Origin 9.0.

## Conflict of Interest

The authors declare no conflict of interest.

## Supporting information

Supporting Information

## Data Availability

The data that support the findings of this study are available from the corresponding author upon reasonable request.
