# Supplementary material for: Stimuli‐Induced Subconformation Transformation of the PSI‐LHCI Protein at Single‐Molecule Resolution
Source: Adv Sci (Weinh). 2023 Apr 28;10(19):2205945. doi: 10.1002/advs.202205945 (PMC10323662; doi:10.1002/advs.202205945)
Supplement: Supplementary file 1 — Supporting Information [file ADVS-10-2205945-s001.pdf]

ADVANCED SCIENCE
Open Access

Supporting Information

for *Adv. Sci.*, DOI 10.1002/advs.202205945

Stimuli-Induced Subconformation Transformation of the PSI-LHCI Protein at Single-Molecule Resolution

Zhiheng Yang, Jie Wang, Bing Yin, Wenzhe Liu, Dongbao Yin, Jianren Shen, Wenda Wang, Lidong Li* and Xuefeng Guo**

Supporting Information

Stimuli-Induced Subconformation transformation of the PSI-LHCI Protein at Single-Molecule Resolution

Zhiheng Yang†[a,b], Jie Wang†[c], Bing Yin[b], Wenzhe Liu[b], Dongbao Yin[b], Jianren Shen[c], Wenda Wang[c], Lidong Li*[a], and Xuefeng Guo*[b,d]*

[a] Z. Yang, Prof. L. Li
State Key Laboratory for Advanced Metals and Materials
School of Materials Science and Engineering, University of Science and Technology Beijing
Beijing, 100083, P. R. China
Email: lidong@mater.ustb.edu.cn

[b] B. Yin, D. Yin, Dr. W. Liu, Prof. X. Guo
Beijing National Laboratory for Molecular Sciences, National Biomedical Imaging Center
College of Chemistry and Molecular Engineering, Peking University
292 Chengfu Road, Haidian District, Beijing 100871, P. R. China
Email: guoxf@pku.edu.cn

[c] J. Wang, Prof. W. Wang
Photosynthesis Research Center, Key Laboratory of Photobiology, Institute of Botany, Chinese Academy of Sciences
Beijing, 100093, P. R. China
Email: wdwang@ibcas.ac.cn

[d] Prof. X. Guo
Center of Single-Molecule Sciences, Institute of Modern Optics, Frontiers Science Center for New Organic Matter
College of Electronic Information and Optical Engineering, Nankai University
38 Tongyan Road, Jinnan District, Tianjin 300350, P. R. China

[†] These authors contributed equally to this work.

WILEY-VCH

Table of Contents

S1. The device fabrication process.

S2. The brief process of mechano–sliding transfer of a SiNW FET array fabrication.

S3. Electrical characterization of SiNW FETs.

S4. The immobilization process of a single PSI-LHCI protein.

S5. STORM characterization of a single PSI-LHCI protein modified SiNW FET device.

S6. The control electrical measurement by the bare SiNW FET Device.

S7. Electrical measurements of the repeated observation by a feat of another SiNW FET Device.

S8. Systematic analysis of statistical data from the vibration process of a single PSI-LHCI protein.

S9. Systematic analysis of statistical data from the adjustment process of a single PSI-LHCI protein responding to the photon flux density.

S10. Control experiments in the photon flux density (1602 μmol •m^{-2}•s^{-1}) but varying the bias voltage from 0.1 V to 0.5 V.

S11. Systematic analysis of statistical data from the adjustment process of a single PSI-LHCI protein responding to the bias voltage intensity.

S12. The occurrence value corresponding to each state at temperature, bias voltage, and photon flux density gradient experiments.

WILEY-VCH

S1. The device fabrication process.

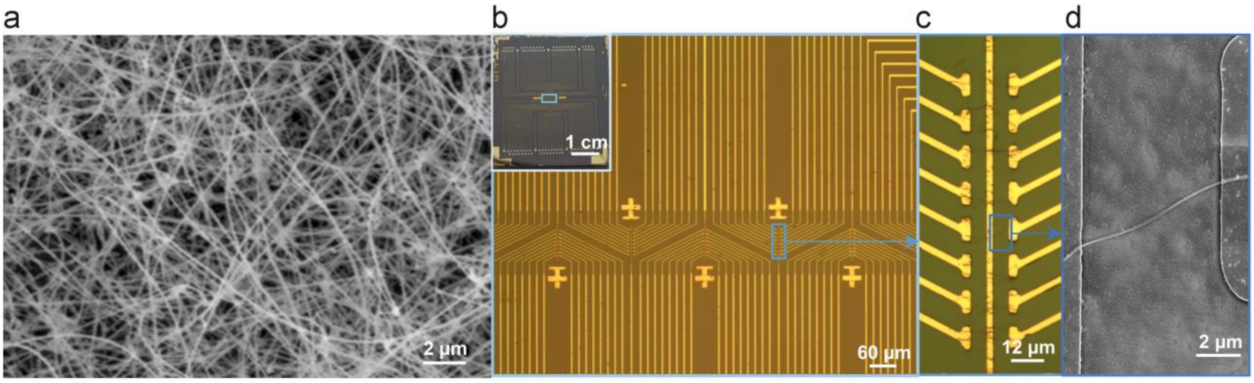

Figure S1. SEM and optical images of a high-density SiNW FET array. (a) SEM image of SiNWs grown by gold-catalyzed CVD. (b) Optical images of SiNW-FET arrays. The inset shows a pattern we designed with 96 pairs of electrodes to make transistor arrays. (c) Magnified optical image of electrode couples. (d) AFM image of a single silicon nanowire between electrode couples.

S2. The brief process of mechano–sliding transfer of a SiNW FET array fabrication.

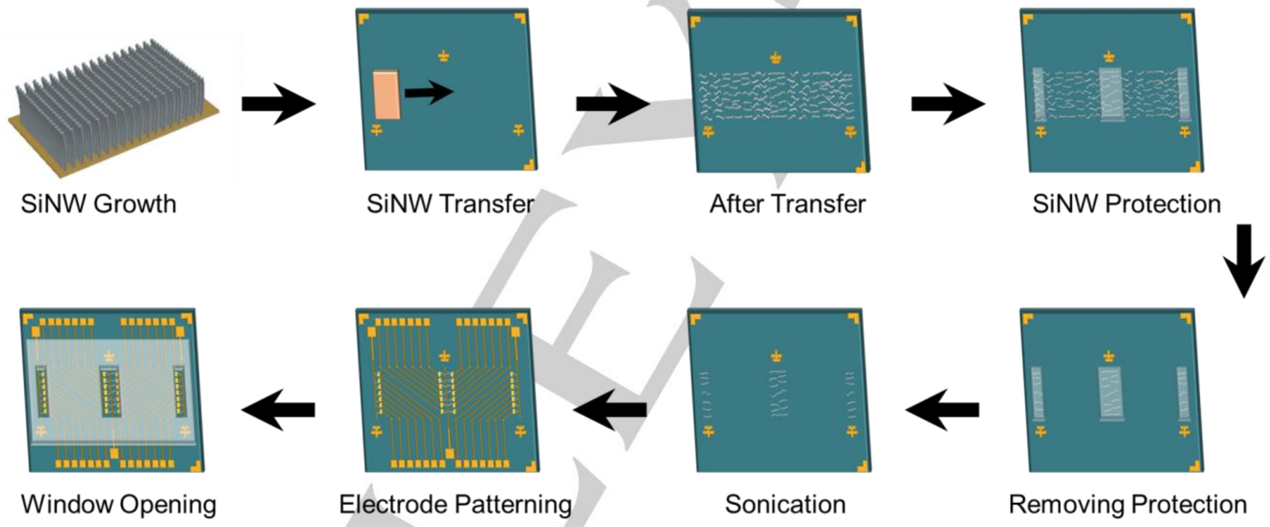

Figure S2. The brief process of mechano–sliding transfer of a SiNW FET array fabrication.

WILEY-VCH

S3. Electrical characterization of SiNW FETs.

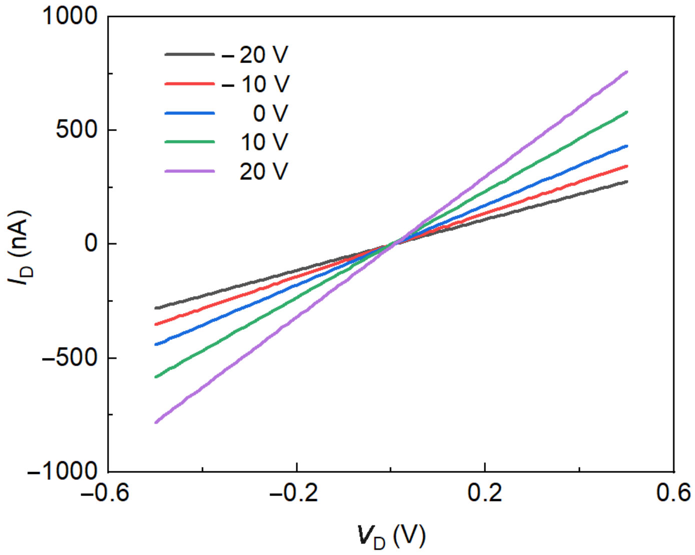

Figure S3. Output characteristics for a *p*-type SiNW FET device at different gate voltages (–20, –10, 0, 10, and 20 V) when the voltage scans from 0.5 V to –0.5 V.

S4. The immobilization process of a single PSI-LHCI protein.

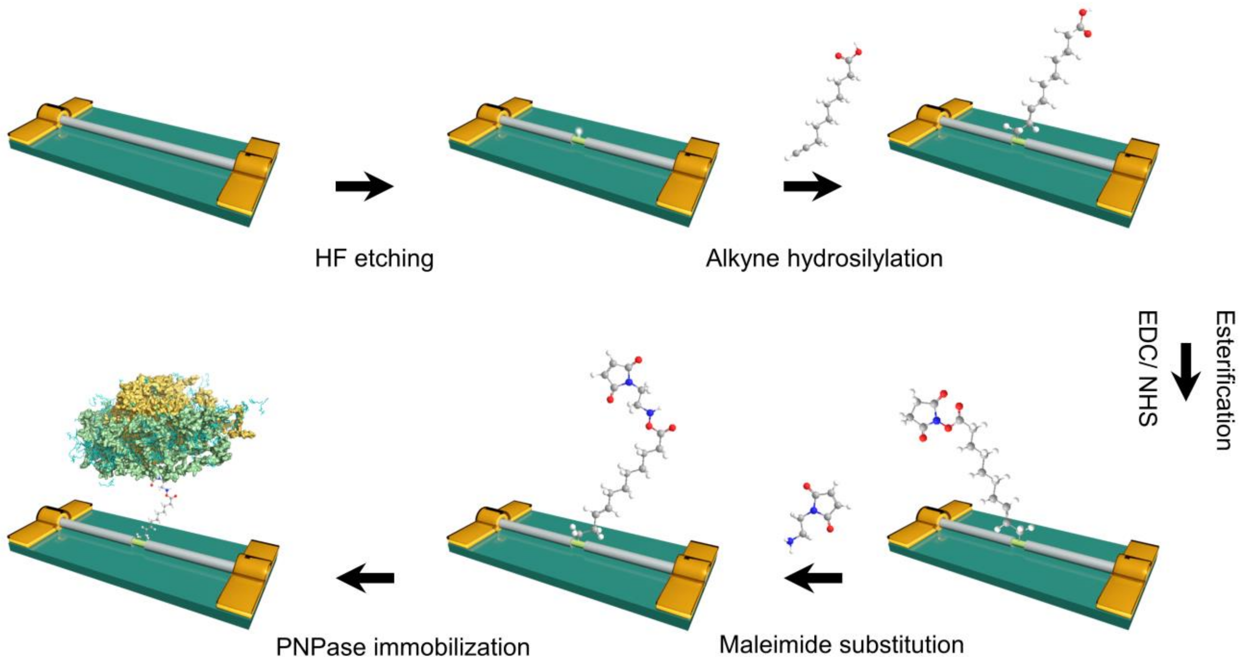

Figure S4. Schematic demonstration of the strategy used for surface functionalization and single PSI-LHRC protein immobilization.

S5. STORM characterization of a single PSI-LHCI protein modified SiNW FET device.

Optical characterization. A buffer liquid layer containing a PSI-LHCI substrate was added to cover the surface of the decorated SiNW device and then attached with a coverslip. Relying on a bright field and laser field (548 nm), the fluorescence signal was monitored by EMCCD through a 100x objective lens. A super-resolution fluorescence microscopy was utilized to capture 5000 photos within a 50 ms exposure. Then, all photos were reconstructed through stochastic optical reconstruction microscopy (STORM) to merge a single-protein photograph and an enlarged image without the background.[1]

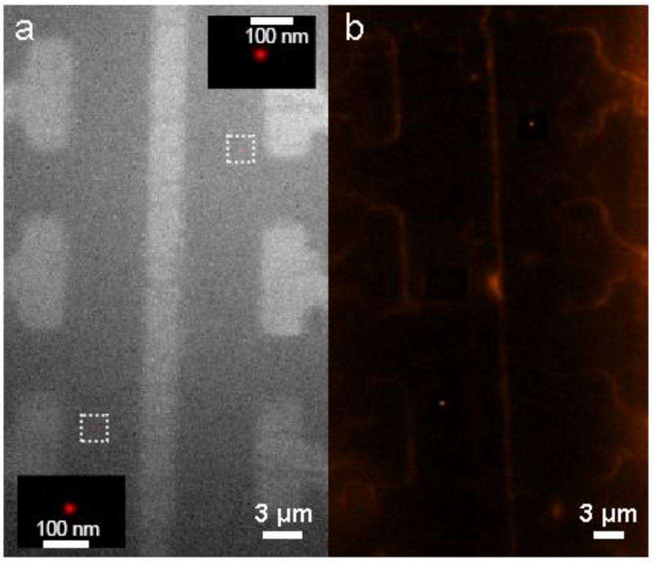

Figure S5. Bright-field (BF) image (a) and STORM image (b) of a single-PSI-LHCI modified device.

WILEY-VCH

S6. The control electrical measurement by the bare SiNW FET Device.

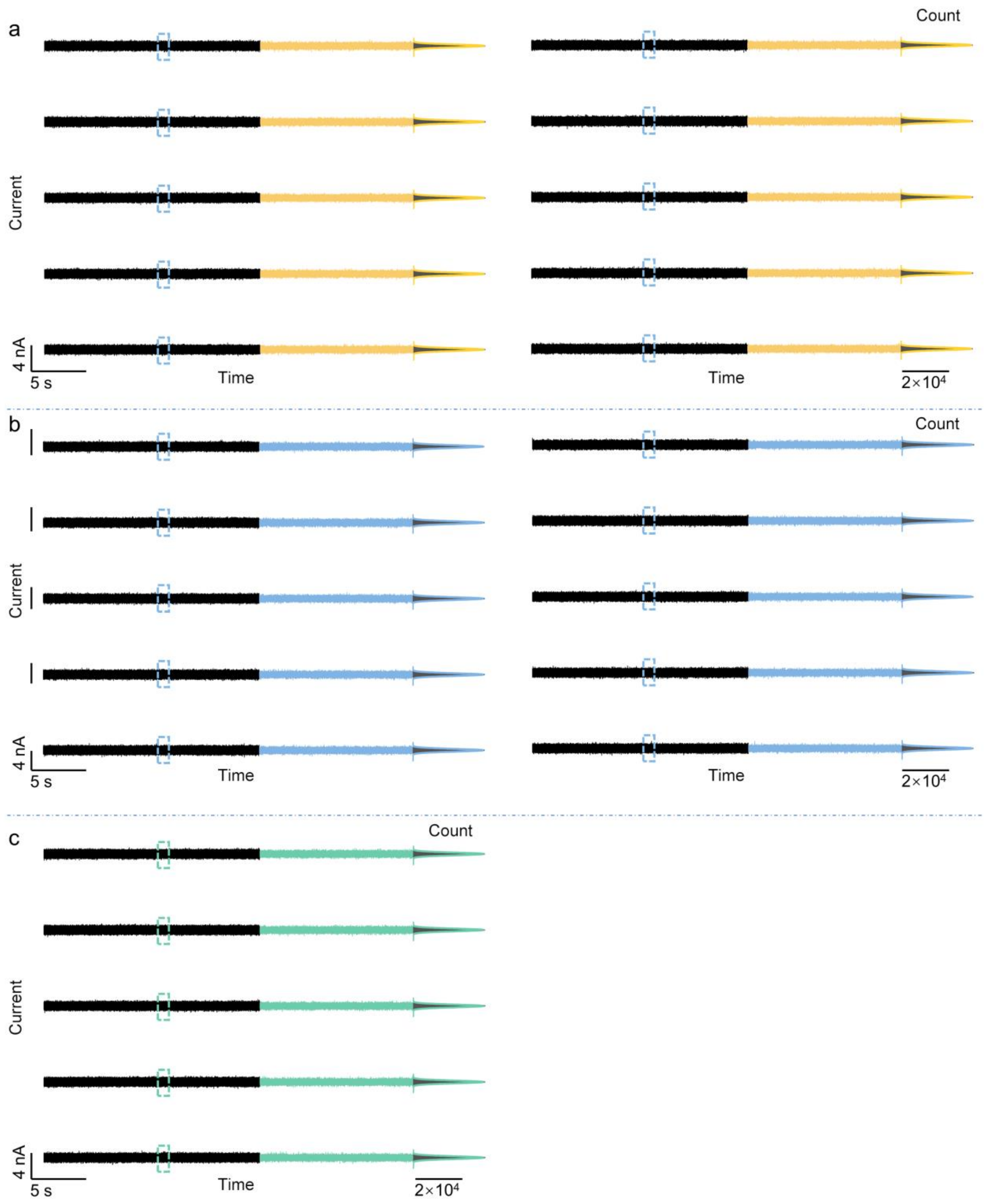

Figure S6. Control electrical measurements of the bare SiNW Device in temperature gradient experiments (top-down: from 17 °C to 37 °C at 5 °C intervals) (a) under dark (left) and light illumination (right), in bias voltage gradient experiments (top-down: from 0.3 V to 1.5 V at 0.3 V intervals) (b) under dark (left) and light illumination (right) and in photon flux density gradient experiments (top-down: from 540 μmol •m^{-2}•s^{-1} to 2700 μmol •m^{-2}•s^{-1} at 540 μmol •m^{-2}•s^{-1} intervals) (c). Electrical trajectories in the middle panel show the 1 s magnified view of each trace (a box drawn with a dashed line) and the corresponding current histograms of 20s electrical trajectories are presented on the right.

S7. Electrical measurements of the repeated observation by a feat of another SiNW FET Device.

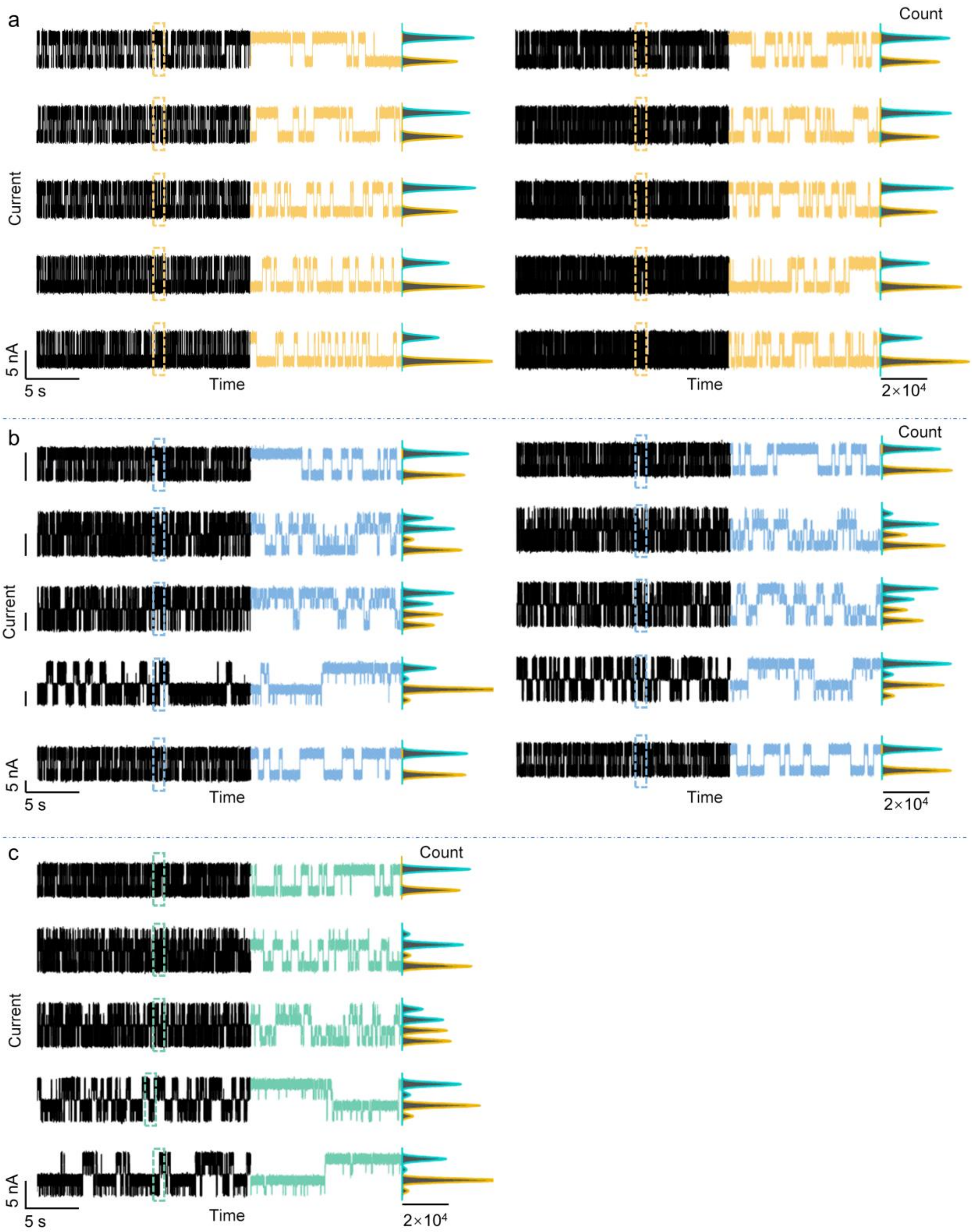

Figure S7. Electrical measurements of the repeated observation by a feat of another SiNW Device in temperature gradient experiments (top-down: from 17 °C to 37 °C at 5 °C intervals) (a) under dark (left) and light illumination (right), in bias voltage gradient experiments (top-down: from 0.3 V to 1.5 V at 0.3 V intervals) (b) under dark (left) and light illumination (right) and in the photon flux density gradient experiments (top-down: from 540 μmol •m^{-2}•s^{-1} to 2700 μmol •m^{-2}•s^{-1} at 540 μmol •m^{-2}•s^{-1} intervals) (c). Electrical trajectories in the middle panel show the 1 s magnified view of each trace (a box drawn with a dashed line) and the corresponding current histograms of 20s electrical trajectories are presented on the right.

S8. Systematic analysis of statistical data from the vibration process of a single PSI-LHCI protein.

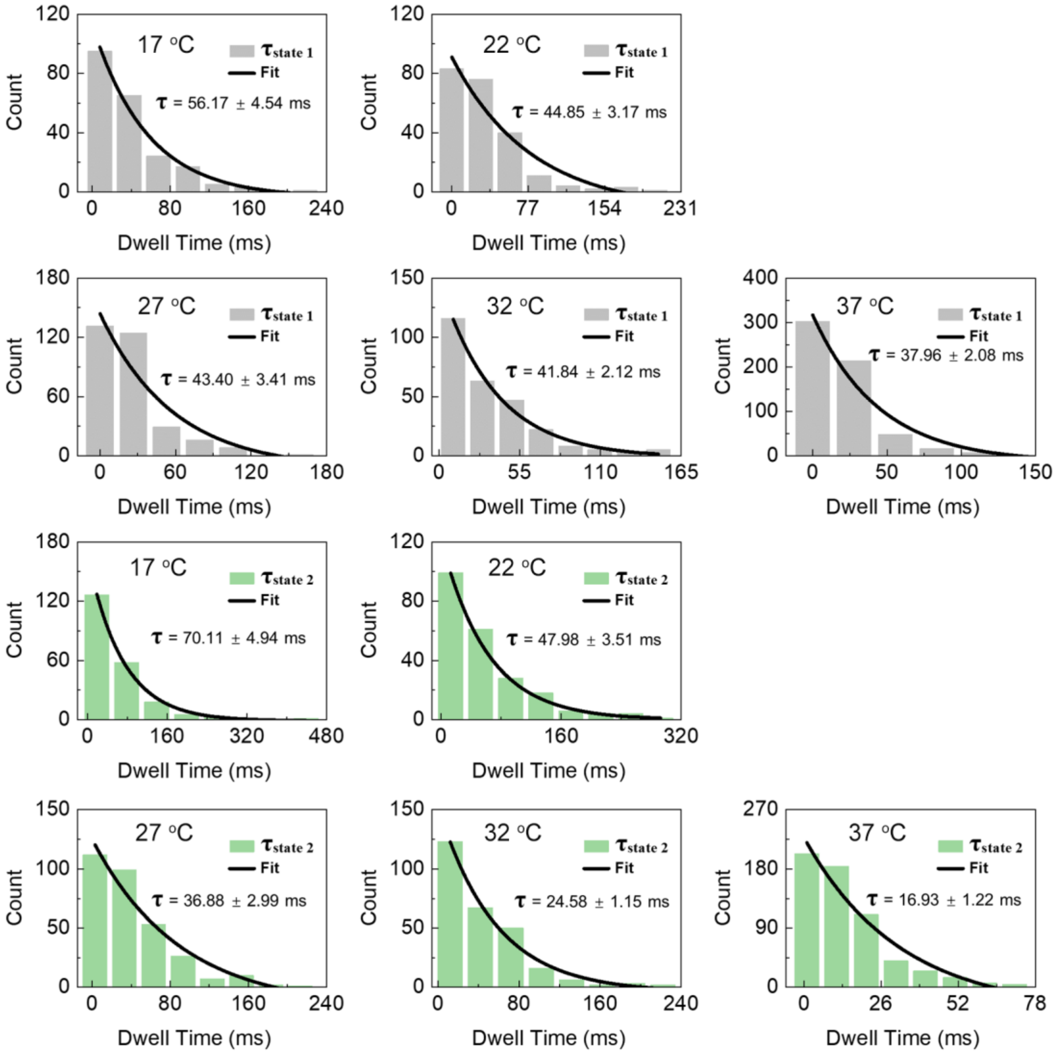

Figure S8. Statistical analyses of the dwell times belonging to State 1 (grey) and State 2 (cyan) in temperature gradient experiments (from upper left to bottom right: from 17 °C to 37 °C at 5 °C intervals) during the vibration process of a single PSI-LHCI protein under dark conditions at a 0.3 V bias voltage.

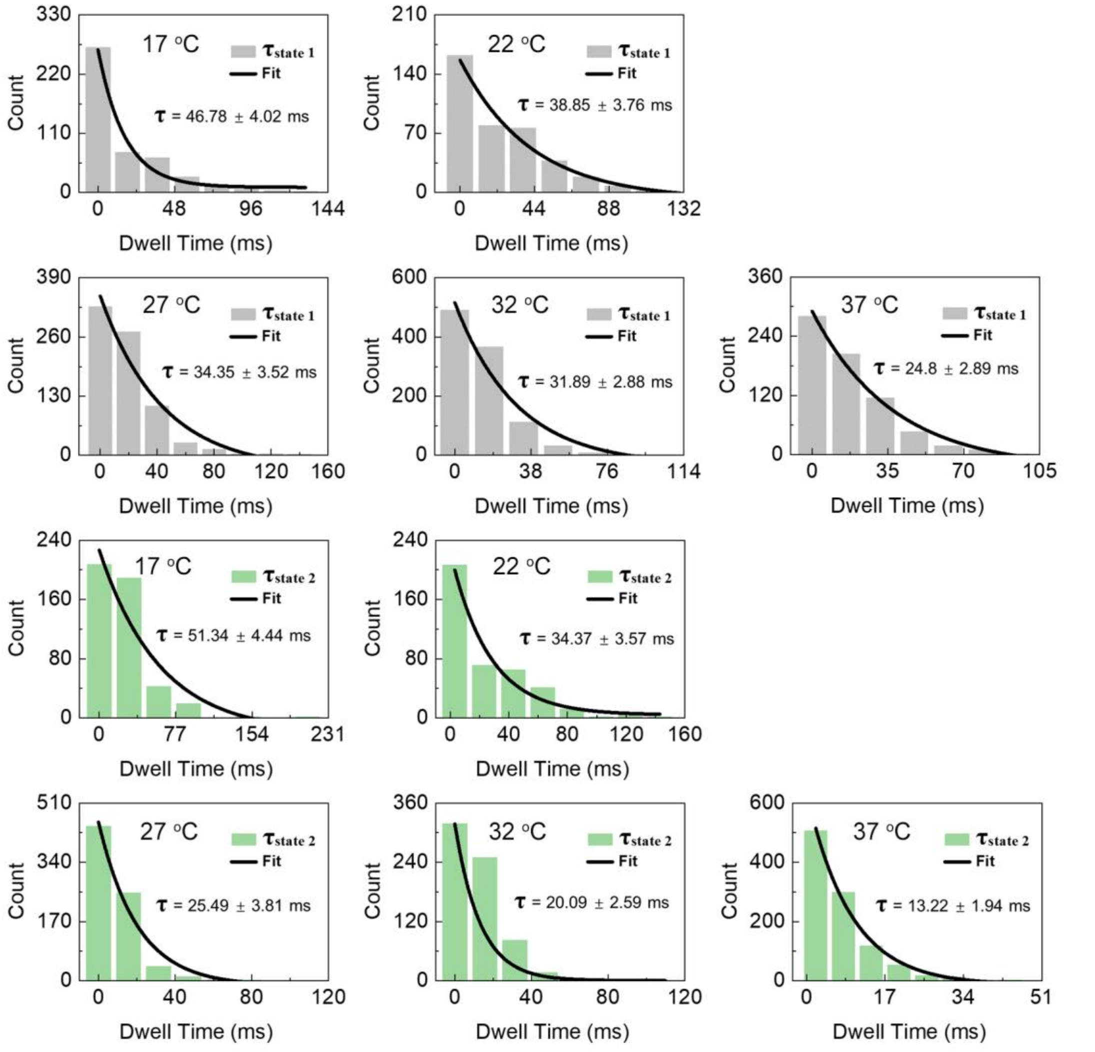

Figure S9. Statistical analyses of the dwell times belonging to State 1 (grey) and State 2 (cyan) in temperature gradient experiments (from upper left to bottom right: from 17 °C to 37 °C at 5 °C intervals) during the vibration process of a single PSI-LHCI protein under light conditions (540 μmol •m^{-2}•s^{-1} photon flux density) at a 0.3 V bias voltage.

WILEY

S9. Systematic analysis of statistical data from the adjustment process of a single PSI-LHCI protein responding to the photon flux density.

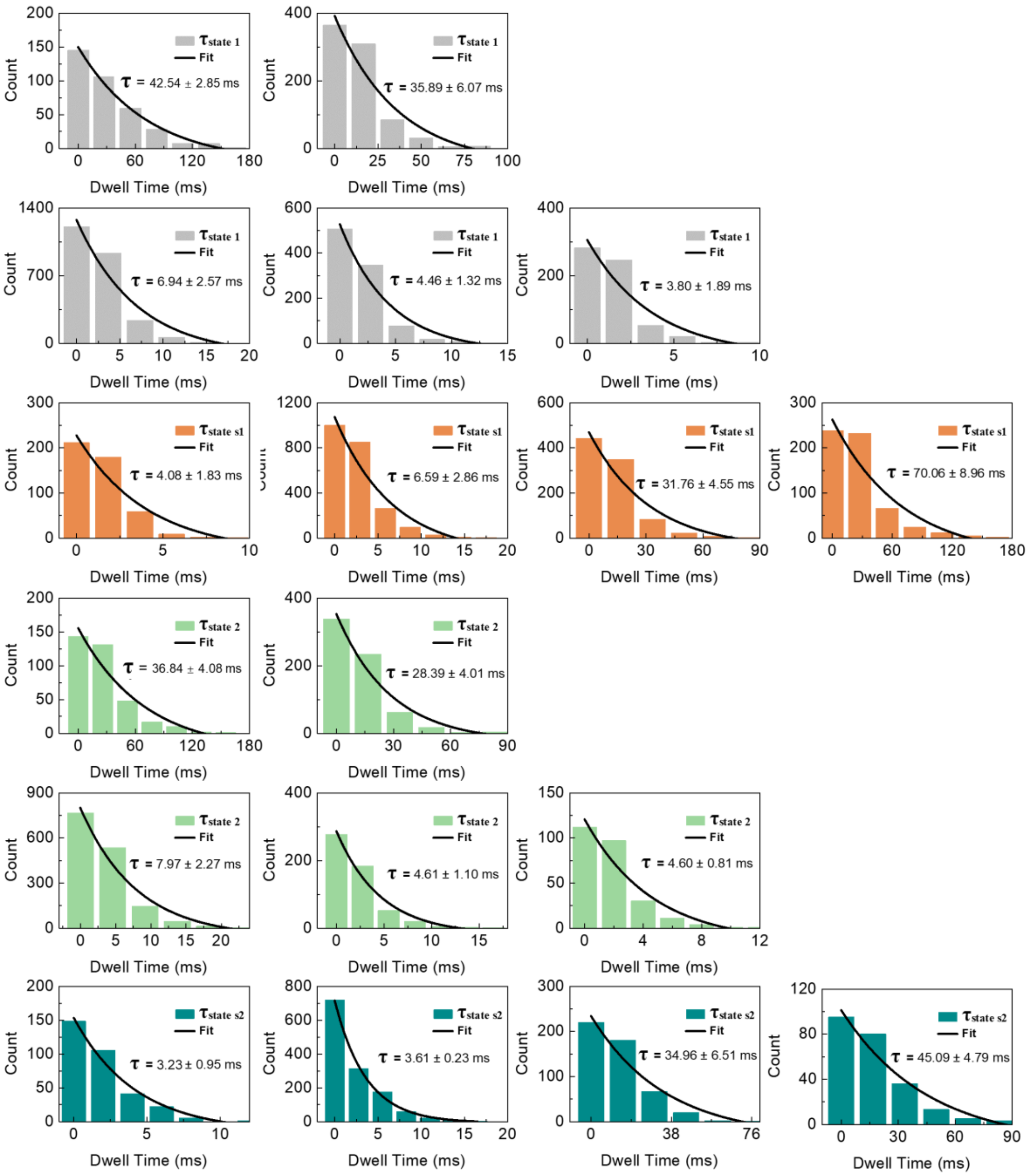

Figure S10. Statistical analyses of the dwell times belonging to State 1 (grey) and State 2 (cyan) in photon flux density gradient experiments (from upper left to bottom right: from 540 μmol •m^{-2}•s^{-1} to 2700 μmol •m^{-2}•s^{-1} at 540 μmol •m^{-2}•s^{-1} intervals) and State s1 (orange) and State s2 (turquoise) in photon flux density gradient experiments (from upper left to bottom right: from 1080 μmol •m^{-2}•s^{-1} to 2700 μmol •m^{-2}•s^{-1} at 540 μmol •m^{-2}•s^{-1} intervals) during the adjustment process of a single PSI-LHCI protein at 0.3 V bias voltage and 22 °C.

S10. Control experiments in the photon flux density (1602 μmol •m^{-2}•s^{-1}) but varying the bias voltage from 0.1 V to 0.5 V.

In order to find out whether the shoulder states responding to the electric field and illumination originate from the same process, we also conducted control experiments under 1602 μmol •m^{-2}•s^{-1} photon flux density by varying the bias voltage from 0.1 V to 0.5 V. The electrical trajectory showed that the occurrence and the dwell time of four states at the photon flux density 1602 μmol •m^{-2}•s^{-1} could be regulated by the bias voltage (Figure S11a). The occurrence and the dwell time of State s1 and State s2 increased gradually, but the tendency belonging to State 1 and State 2 was inverse (Figures S11b and c). The response trend was consistent with that under photon flux density gradient, suggesting that the shoulder states induced by the illumination and electric field have uniform structures and the PSI-LHCI has a consistent response mechanism to both illumination and electric field. Relying on the Stark fluorescence spectroscopy, the contribution of charge-transfer states to the quenching is early revealed inside LHCII and the charge-transfer interactions between chlorophyll and chlorophyll (also between chlorophyll and carotenoid) coupled to the chlorophyll exciton state in the terminal emitter locus are also proposed.[2] Therefore, our electrical data obtained through collaborative measurements combining illumination and electric field gradient conditions suggest that charge transfer among chlorophylls also happened inside LHCI.

WILEY-VCH

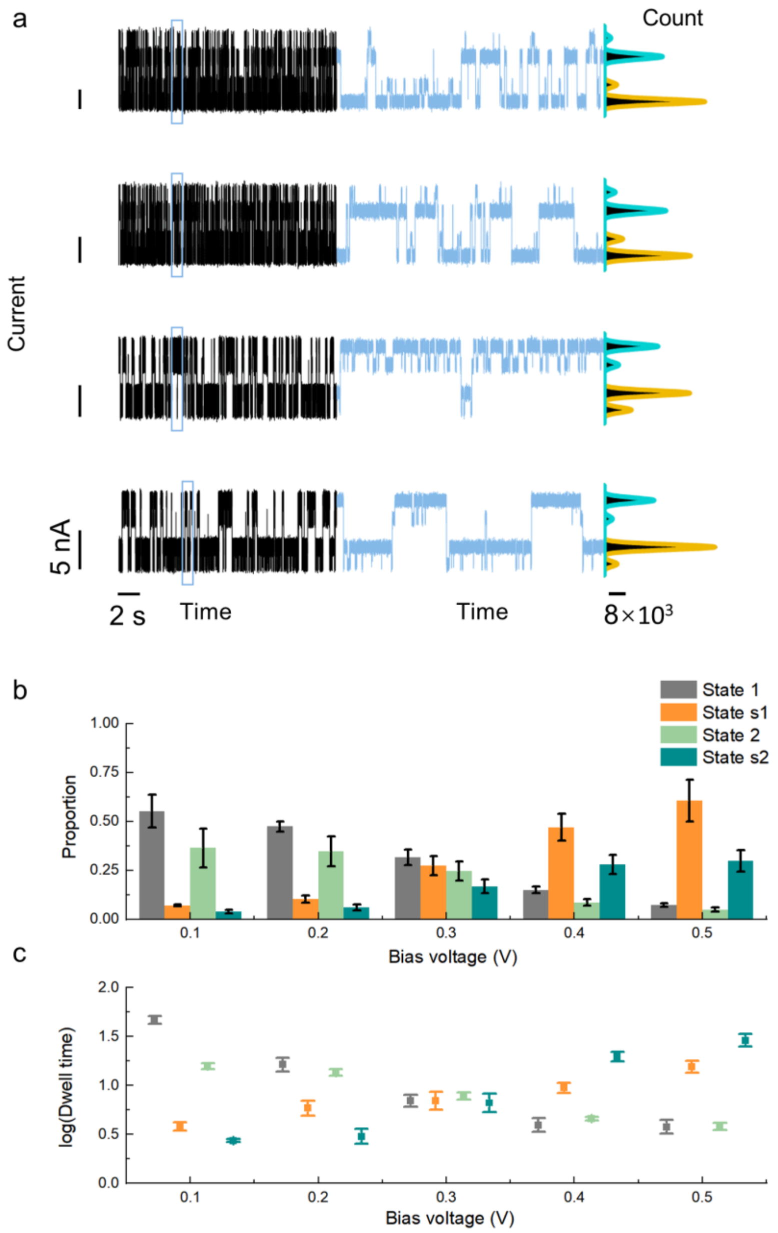

Figure S11. Control experiments in the photon flux density 1602 μmol •m^{-2}•s^{-1} but varying the bias voltage from 0.1V to 0.5V. a) The electrical data of control experiments from top to down (0.1V, 0.2V, 0.4V, 0.5V). b) The distribution of the proportion belonging to each state from 0.1V to 0.5V. c) The distribution of the dwell time belonging to each state from 0.1V to 0.5V.

S11. Systematic analysis of statistical data from the adjustment process of a single PSI-LHCI protein responding to the bias voltage intensity.

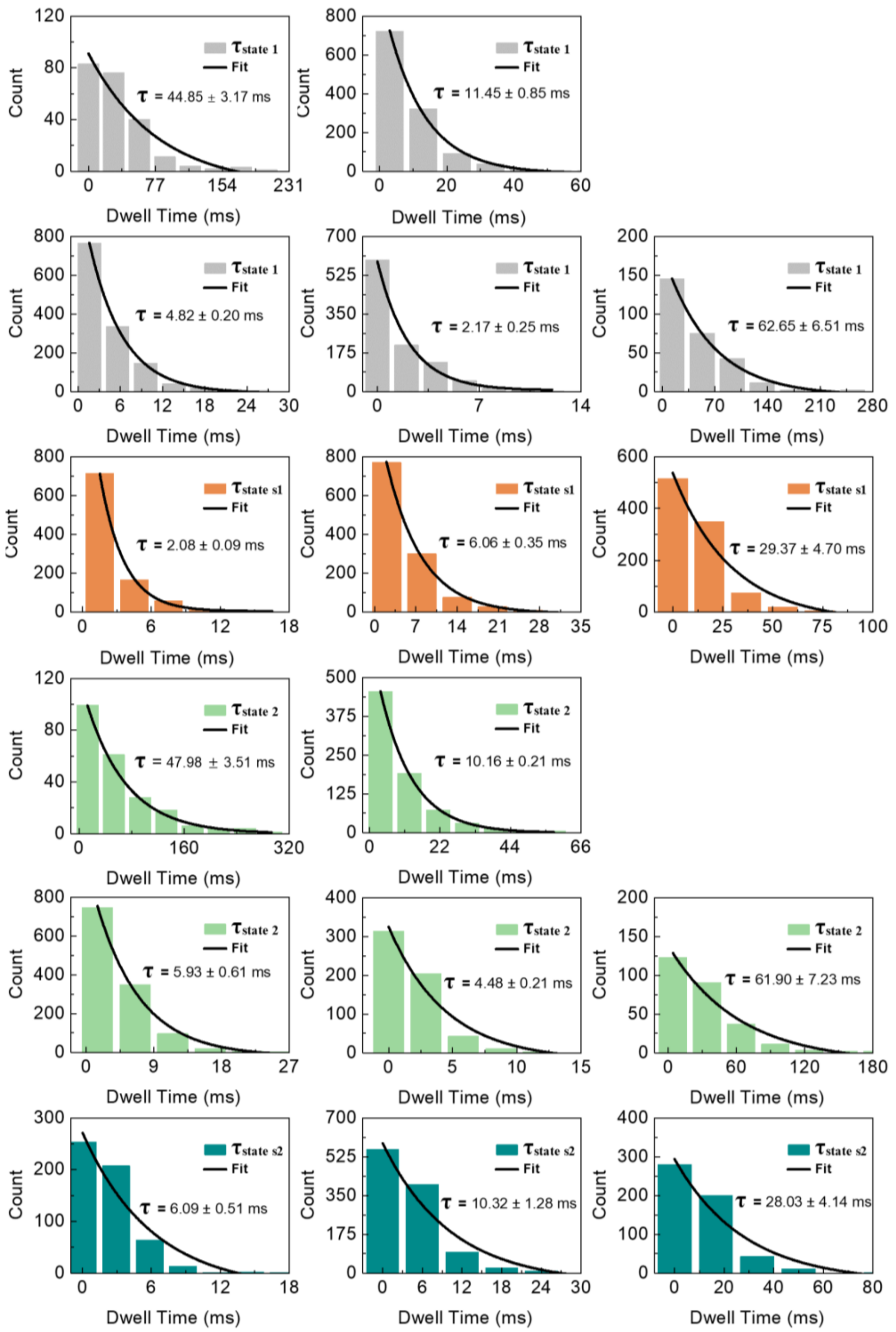

Figure S12. Statistical analyses of the dwell times belonging to State 1 (grey) and State 2 (cyan) in bias voltage gradient experiments (from upper left to bottom right: from 0.3 V to 1.5 V at 0.3 V intervals) and State s1 (orange) and State s2 (turquoise) in bias voltage gradient experiments (from upper left to bottom right: from 0.6 V to 1.2 V at 0.3 V intervals) during the adjustment process of a single PSI-LHCI protein under dark at 22 ^{o}C.

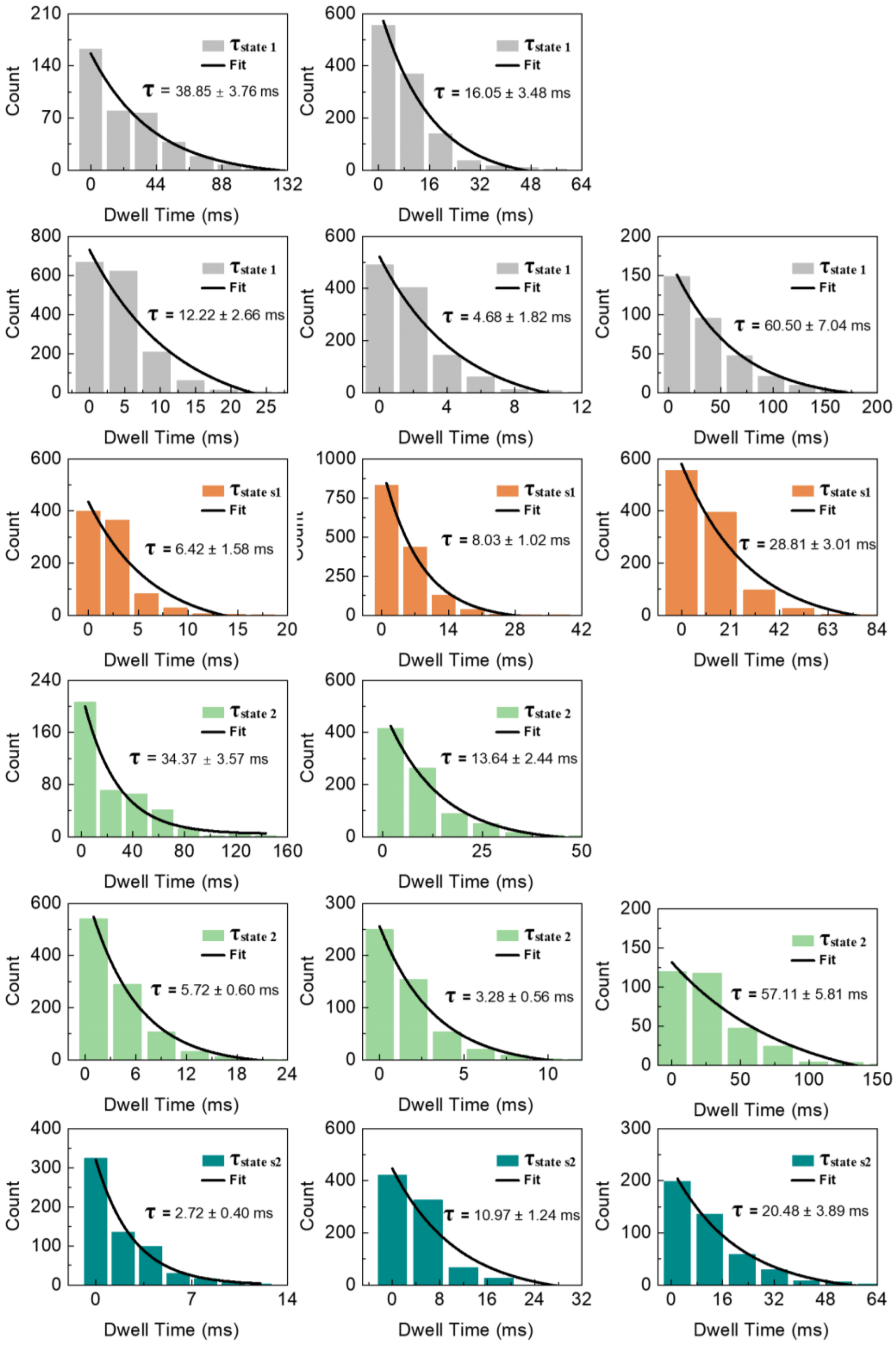

Figure S13. Statistical analyses of the dwell times belonging to State 1 (grey) and State 2 (cyan) in bias voltage gradient experiments (from upper left to bottom right: from 0.3 V to 1.5 V at 0.3 V intervals) and State s1 (orange) and State s2 (turquoise) in bias voltage gradient experiments (from upper left to bottom right: from 0.6 V to 1.2 V at 0.3 V intervals) during the adjustment process of a single PSI-LHCI protein under the light conditions (540 μmol •m^{-2}•s^{-1} photon flux density) at 22 °C.

S12. Table S1 the occurrence value corresponding to each state at temperature, bias voltage, and photon flux density gradient experiments.

Temperature (℃)	State 1 (%)/Dark	State 2 (%)/Dark	State 1 (%)/Light	State 2(%)/Light
17	35.65 ± 8.56	64.35 ± 5.85	29.64 ± 1.48	70.36 ± 7.89
22	42.17 ± 3.61	57.83 ± 9.12	37.80 ± 3.58	62.20 ± 0.18
27	49.10 ± 1.05	50.90 ± 9.17	46.75 ± 8.47	53.25 ± 7.60
32	60.23 ± 7.68	39.77 ± 6.38	58.15 ± 8.81	41.85 ± 5.53
37	67.09 ± 2.30	32.91 ± 4.83	65.57 ± 4.22	34.43 ± 9.11

Bias Voltage (V)	0.3	0.6	0.9	1.2	1.5
State 1 (%)/Dark	42.17 ± 9.17	49.63 ± 4.02	26.77 ± 2.73	9.06 ± 0.80	60.90 ± 3.15
State s1 (%)/Dark		10.57 ± 2.35	27.76 ± 2.55	58.01 ± 9.11	
State 2 (%)/Dark	57.83 ± 3.068	33.82 ± 0.20	23.25 ± 3.70	5.22 ± 3.65	39.09 ± 5.86
State s2 (%)/Dark		5.98 ± 1.85	22.21 ± 5.00	27.69 ± 3.38	
State 1 (%)/Light	37.80 ± 4.32	47.78 ± 1.15	32.03 ± 1.69	9.94 ± 2.65	57.58 ± 7.67
State s1 (%)/Light		9.84 ± 3.41	32.79 ± 0.64	60.68 ± 8.09	
State 2 (%)/Light	62.20 ± 5.52	35.98 ± 6.60	17.89 ± 1.09	4.34 ± 1.91	42.67 ± 2.23
State s2 (%)/Light		6.40 ± 5.06	17.29 ± 3.76	25.04 ± 6.77	

Photon flux density (μ mol •m^{-2}•s^{-1})	540	1080	1620	2160	2700
State 1 (%)	44.17 ± 3.43	39.35 ± 3.36	31.54 ± 3.98	8.86 ± 6.06	4.19 ± 4.62
State s1 (%)		3.10 ± 2.91	27.18 ± 4.89	54.69 ± 4.53	73.84 ± 5.48
State 2 (%)	55.83 ± 3.86	54.32 ± 4.84	24.51 ± 4.88	5.66 ± 3.65	2.23 ± 1.77
State s2 (%)		3.24 ± 2.08	16.77 ± 3.53	30.80 ± 3.61	19.74 ± 3.10

References

[1] C. Yang, L. Zhang, C. Lu, C. X. S. Zhou, X. Li, Y. Li, Y. Yang, Y. Li, Z. Liu, J. Yang, K. N. Houk, F. Mo, X. Guo, *Nat. Nanotechnol.* **2021**, *16*, 1214.

[2] M. Wahadoszamen, R. Berera, A. M. Ara, E. Romero, R. van Grondelle, *Phys. Chem. Chem. Phys.* **2012**, *14*, 759.

WILEY-VCH
